# The relationship between prostatic microvessel density and different concentrations of oestrogen/androgen in Sprague-Dawley rats

**DOI:** 10.1186/s40001-022-00719-7

**Published:** 2022-06-07

**Authors:** Bo Wang, Di Pan, Yong Ban, Zhaolin Sun, Ye Tian, Guangheng Luo

**Affiliations:** 1 Department of Urology, Guizhou Provincial People’s Hospital, Guiyang, Guizhou People’s Republic of China; 2 Institute of Medical Microbiology and Hygiene, Medical Center University of Freiburg, Freiburg, Germany

**Keywords:** Oestrogen, Androgen, Prostatic microvessel density

## Abstract

**Background:**

Currently, there are relatively few studies on the effects of changes in oestrogen and androgen levels on prostatic microvessel density (MVD). This article aimed to study the changes in prostatic MVD in Sprague-Dawley (SD) rats after castration under the effect of oestrogen/androgen at different concentrations.

**Methods:**

Male SD rats aged 3–4 months were randomly divided into a control group, a castration group, and groups with different concentrations of oestrogen/androgen treatment after castration. Dihydrotestosterone (DHT) and oestradiol (E) were administered daily by subcutaneous injection for one month. All the rats were killed by cervical dislocation after one month, and the serum DHT and E concentrations of the rats in each group were measured by ELISA. Prostate tissue specimens were immunohistochemically stained with monoclonal antibodies against CD34 and factor VIII for MVD.

**Results:**

Compared with the control group, the MVD decreased significantly in the castration group (*P* < 0.05). When the exogenous E concentration was constant, in general, the MVD of rats in all the groups increased with increasing exogenous DHT concentration. Compared with the castration group, the MVD increased significantly in the E0.05 + DHT0.015 mg/kg, E0.05 + DHT0.05 mg/kg, E0.05 + DHT0.15 mg/kg, E0.05 + DHT0.5 mg/kg, and E0.05 + DHT1.5 mg/kg groups (*P* < 0.05). In addition, when the exogenous DHT concentration was constant, the MVD increased with increasing exogenous E concentration in all the groups. Among them, compared with the control and castration groups, the MVD increased significantly in the DHT0.15 + E0.015 mg/kg, DHT0.15 + E0.15 mg/kg, and DHT0.15 + E0.5 mg/kg groups (*P* < 0.05).

**Conclusions:**

Androgens play an important role in the regulation of prostatic MVD in SD rats, and a decrease in DHT concentration can induce a decrease in prostatic MVD. In contrast, prostatic MVD can be increased with increasing DHT concentration. In addition, prostatic MVD can be increased gradually with increasing oestrogen concentration.

## Background

Transurethral resection of the prostate (TURP) is currently one of the major treatments for benign prostatic hyperplasia (BPH) [1]. Bleeding constitutes one of the complications of TURP, and its cause has attracted increasing attention. Increased bleeding during TURP has been found to be associated with increased prostate volume and increased angiogenesis in prostate tissue [2]. Lekås et al. [3] found that castration reduced the number and proliferation rate of endothelial cells in the ventral prostate of adult rats. However, after testosterone supplementation with androgen, the number and proliferation rate of endothelial cells can be restored to normal levels. In addition, testosterone treatment can rapidly normalize ventral prostatic blood flow in adult rats [4]. Therefore, these studies suggested that androgen can directly or indirectly regulate the vasculature in rat prostate tissue.

However, we noted that normal males have a certain concentration of oestrogen, and in the prostate epithelium and stroma, there are a large number of oestrogen receptors, the activation of which also profoundly affects the development and progression of prostate diseases [5]. Oestrogen induces remodelling of the collateral vasculature and may stimulate the growth of resistance vessels, thereby providing protection during the development of coronary artery occlusion [6]. In addition, administration of oestrogen in vitro or in vivo can promote the proliferation and migration of endothelial cells, thereby promoting the formation of new blood vessels [7]. Therefore, it is reasonable to suspect that oestrogen also has an important role in the regulation of prostate microvascular density.

In previous studies, it has been shown that the maintenance of a dynamic balance between oestrogen and androgen levels contributes to the normal growth and development of the prostate gland and the maintenance of its physiological functions, while disruption of this balance can lead to the proliferation and apoptosis of prostate cells and consequently to the development of BPH [8]. However, there are relatively few studies on the effects of changes in oestrogen and androgen levels on prostatic MVD. When the concentration of exogenous oestrogens and/or androgens is altered, this causes a change in the concentration ratio of oestrogen/androgen, which in turn disrupts the hormonal balance in the body. It remains unknown whether prostate microvascular density changes in response to the altered concentrations during this process. Therefore, this article aimed to study the changes in prostatic MVD in SD rats after castration under the effect of oestrogen/androgen at different concentrations and to provide a new theoretical basis for revealing the influence of sex hormone levels on prostatic MVD.

The endothelial antigen CD34 was chosen because it is recognized as a direct marker of the degree of vascularization and neoangiogenesis [9]. In addition, factor VIII can be used because it is localized in vascular endothelial cells [10]. Immunohistochemical staining is performed using a specific vascular endothelial monoclonal antibody against specific vascular endothelial components (cell adhesion molecule CD34 or factor VIII antigen) to display microvascular components of tissues and calculate MVD values. Therefore, both CD34 and factor VIII can be used to assess MVD, with successful application reported by Bettencourt et al. [11] and Gettman et al. [12].

## Methods

### Experimental animals

Fifty-two male SD rats, aged 3–4 months, weighing 250–350 g, were provided by the Experimental Animal Center of Guizhou Medical University, Licence no. SCXK (Guizhou) 2012–0001. The animals were handled in accordance with the "Opinions under the Guidance of Treating Experimental Animals". All the animals (2 rats in each cage) were housed at a room temperature of 20–26 °C and a relative humidity of 40–70% during a light cycle from 8:00 to 20:00. During the feeding period, the animals were free to access water and food (soy-free rodent diet). This experiment was approved by the Ethics Committee of Guizhou Provincial People’s Hospital (No. 2018025).

### Surgery and experimental groups

SD rats were castrated by removing both testes through the scrotal approach under general anaesthesia. The rats were divided into 14 groups with 4 rats in each group; the E0.05 + DHT0.15 mg/kg group and the DHT0.15 + E0.05 mg/kg group were the same group. The experimental protocol is shown in Table 1. DHT and E were administered daily by subcutaneous injection for one month [13, 14].

**Table 1:** Structure of the experiment

Group	Surgery	Agents and doses	*n*
Control group	Non-castrated	Corn oil	4
Castration group	Castrated	Corn oil	4
E0.05 + DHT			
0	Castrated	E0.05 mg/kg + DHT0 mg/kg	4
0.015	Castrated	E0.05 mg/kg + DHT0.015 mg/kg	4
0.05	Castrated	E0.05 mg/kg + DHT0.05 mg/kg	4
0.15	Castrated	E0.05 mg/kg + DHT0.15 mg/kg	4
0.5	Castrated	E0.05 mg/kg + DHT0.05 mg/kg	4
1.5	Castrated	E0.05 mg/kg + DHT1.5 mg/kg	4
DHT0.15 + E			
0	Castrated	DHT0.15 mg/kg + E0 mg/kg	4
0.005	Castrated	DHT0.15 mg/kg + E0.005 mg/kg	4
0.015	Castrated	DHT0.15 mg/kg + E0.015 mg/kg	4
0.05	Castrated	DHT0.15 mg/kg + E0.05 mg/kg	4
0.15	Castrated	DHT0.15 mg/kg + E0.15 mg/kg	4
0.5	Castrated	DHT0.15 mg/kg + E0.5 mg/kg	4

### Detection of serum E and DHT levels in each group of SD rats

Twenty-four hours after the last subcutaneous injection, all the rats were killed by cervical dislocation. After the abdominal aorta was fully exposed, approximately 5–10 mL of blood was collected from the abdominal aorta using a preheparinized 10-mL syringe. The blood specimen was placed in a centrifuge tube for 15 min at room temperature and then incubated in a refrigerator at 4 °C for 2 h. The blood specimen was then removed and centrifuged at 3000 r/min for 3 min at room temperature, and the supernatant was removed and stored in a refrigerator at − 20 °C. The serum E and DHT concentrations were measured according to the ELISA kit instructions (China Shanghai Hufeng Chemical Company).

### Detection of the MVD of the prostate tissue in each group of SD rats

The immunohistochemical method was used to detect the expression levels of CD34 and factor VIII in the prostate tissue of SD rats in each group. Five areas of each specimen were randomly selected, and the numbers of CD34- and factor VIII-positive cells in each high-power field of vision were counted to calculate the positive incidence of CD34 and factor VIII by using a light microscope at a magnification of 400× (positive incidence = the number of positive cells/the total number of cells × 100%). Furthermore, the mean score of CD34- and factor VIII-positive cells was calculated in each specimen to obtain the MVD [15].

### Statistical methods

The experimental data were statistically processed by SPSS 24.0 software. Normally distributed data are shown as mean ± SD and are presented by the line graph. Non‐normally distributed data are shown as medians with interquartile ranges and presented by the box-and-whisker plots. Nonparametric tests and one-way analysis of variance were used to analyse the results. A value of *P* < 0.05 was considered statistically significant.

## Results

### Changes in serum DHT and E concentrations in each group

**Fig. 1:**
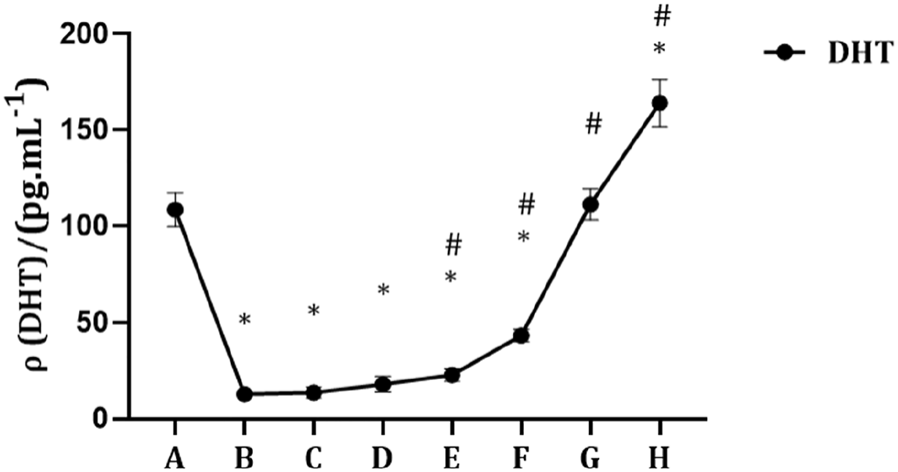
Changes in serum DHT concentrations in SD rats. The data are shown as mean ± SD, and the statistical difference was determined using one-way analysis of variance (*n* = 4). *P < 0.05 compared to control group, ^#^*P* < 0.05 compared to castration group. **A** Control group. **B** Castration group. **C** E0.05 + DHT0 mg/kg group. **D** E0.05 + DHT0.015 mg/kg group. **E** E0.05 + DHT0.05 mg/kg group. **F** E0.05 + DHT0.15 mg/kg group. **G** E0.05 + DHT0.5 mg/kg group. **H** E0.05 + DHT1.5 mg/kg group

As shown in Fig. 1, compared with the control group, the serum DHT concentration significantly decreased in the castration group (*P* < 0.05). When the concentration of exogenous E was constant, the serum DHT concentration increased with increasing exogenous DHT concentration. In addition, compared with the castration group, the serum DHT concentration significantly increased in the E0.05 + DHT0.05 mg/kg, E0.05 + DHT0.15 mg/kg, E0.05 + DHT0.5 mg/kg, and E0.05 + DHT1.5 mg/kg groups (*P* < 0.05).

**Fig. 2:**
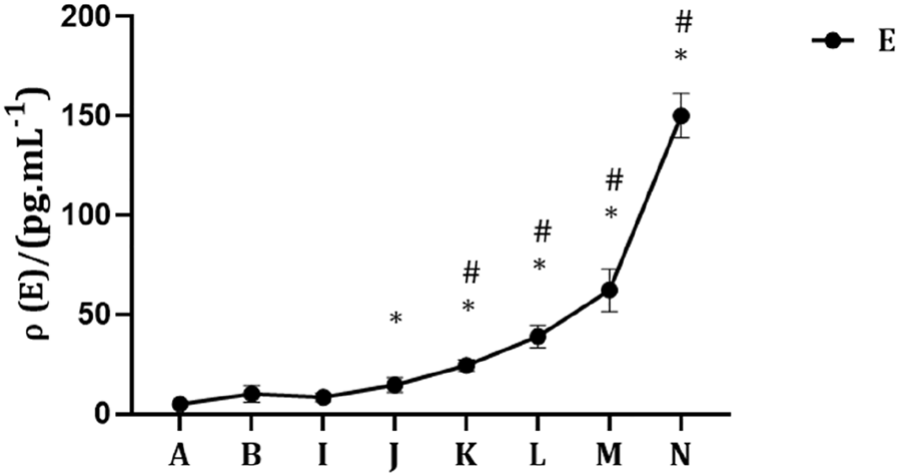
Changes in serum E concentrations in SD rats. The data are shown as mean ± SD, and the statistical difference was determined using one-way analysis of variance (*n* = 4). *P < 0.05 compared to control group, ^#^*P* < 0.05 compared to castration group. **A** Control group. **B** Castration group. **I** DHT0.15 + E0 mg/kg group. **J** DHT0.15 + E0.005 mg/kg group. **K** DHT0.15 + E0.015 mg/kg group. **L** DHT0.15 + E0.05 mg/kg group. **M** DHT0.15 + E0.15 mg/kg group. **N** DHT0.15 + E0.5 mg/kg group

As shown in Fig. 2, when the exogenous DHT concentration was constant, the serum E concentration increased with increasing exogenous E concentration. Furthermore, compared with the control and castration groups, the serum E concentration increased significantly in the DHT0.15 + E0.015 mg/kg, DHT0.15 + E0.05 mg/kg, DHT0.15 + E0.15 mg/kg and DHT0.15 + E0.5 mg/kg groups (*P* < 0.05).

### Expression of CD34 and factor VIII in each group

**Fig. 3:**
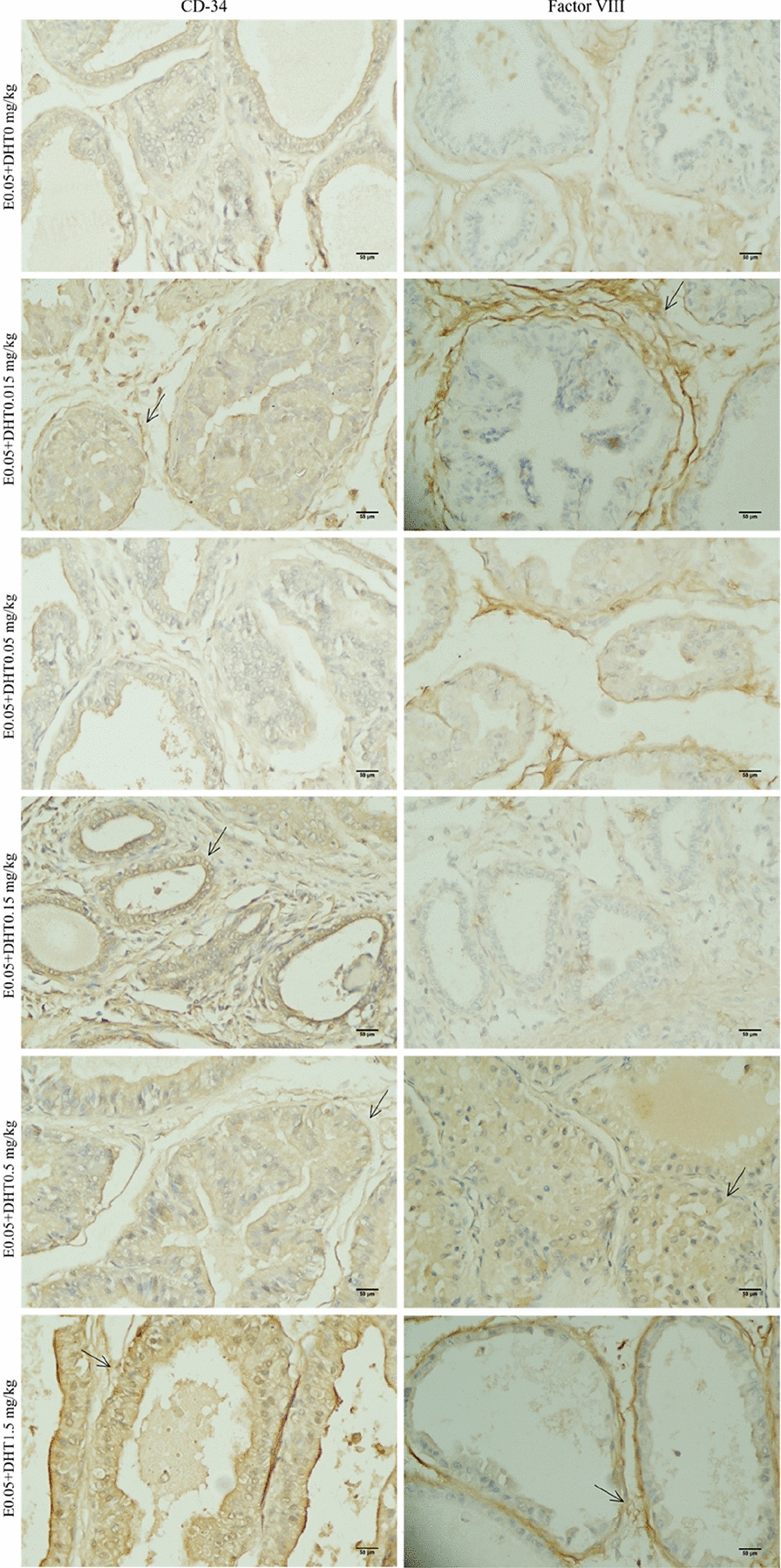
The expressions of CD34 and factor VIII in different DHT concentration groups. IHC × 400

**Fig. 4:**
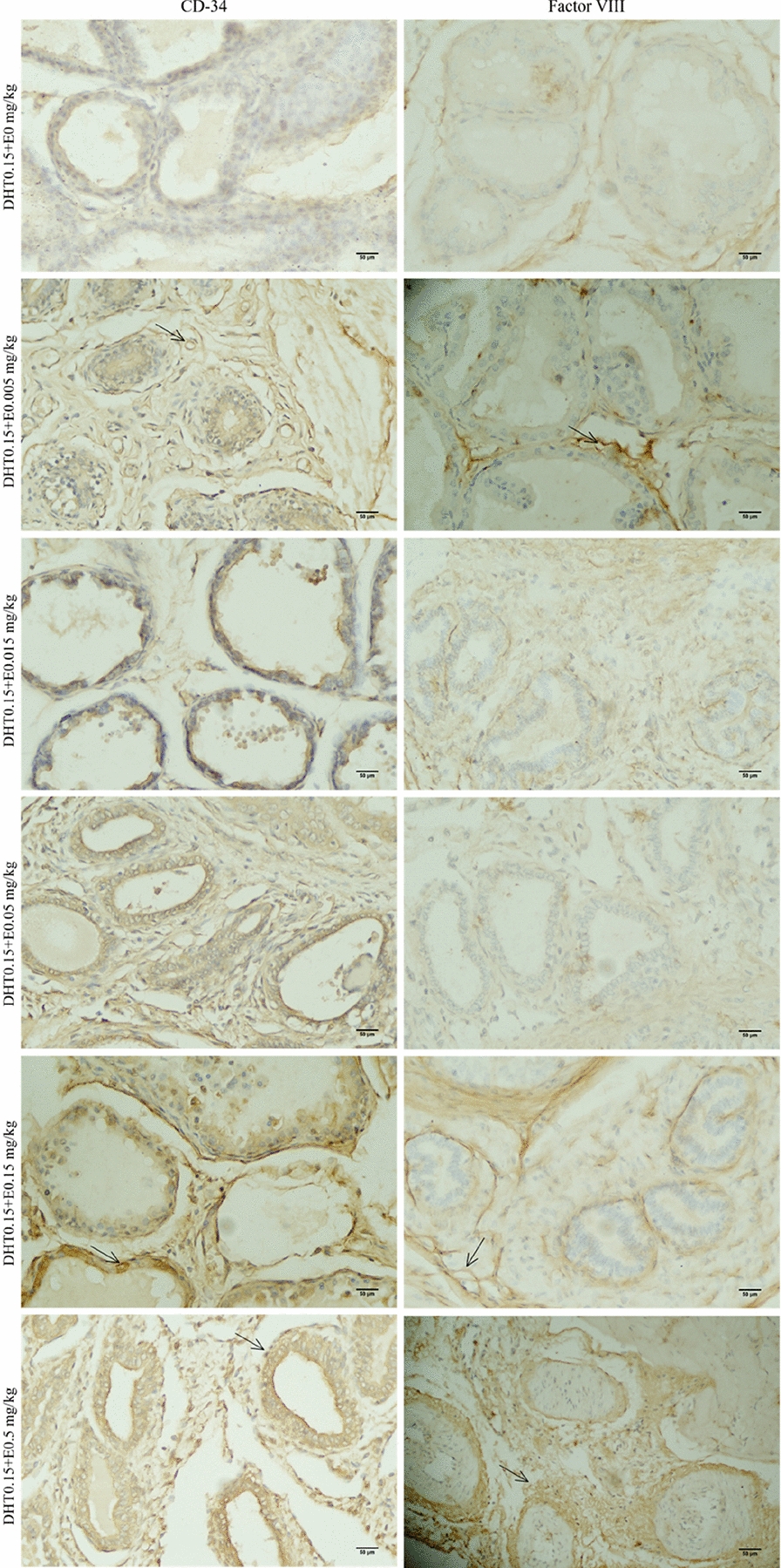
The expressions of CD34 and factor VIII in different E concentration groups. IHC × 400

The expression of CD34 and factor VIII in the prostate tissue of SD rats in each group was detected by immunohistochemistry (Figs. 3 and 4, control and castration groups are not shown), and the quantitative analysis showed that the positive rate of CD34 in the castration group was significantly decreased compared with the control group (*P* < 0.05). However, the positive rate of factor VIII increased compared with the control group, but the difference was not statistically significant.

**Fig. 5:**
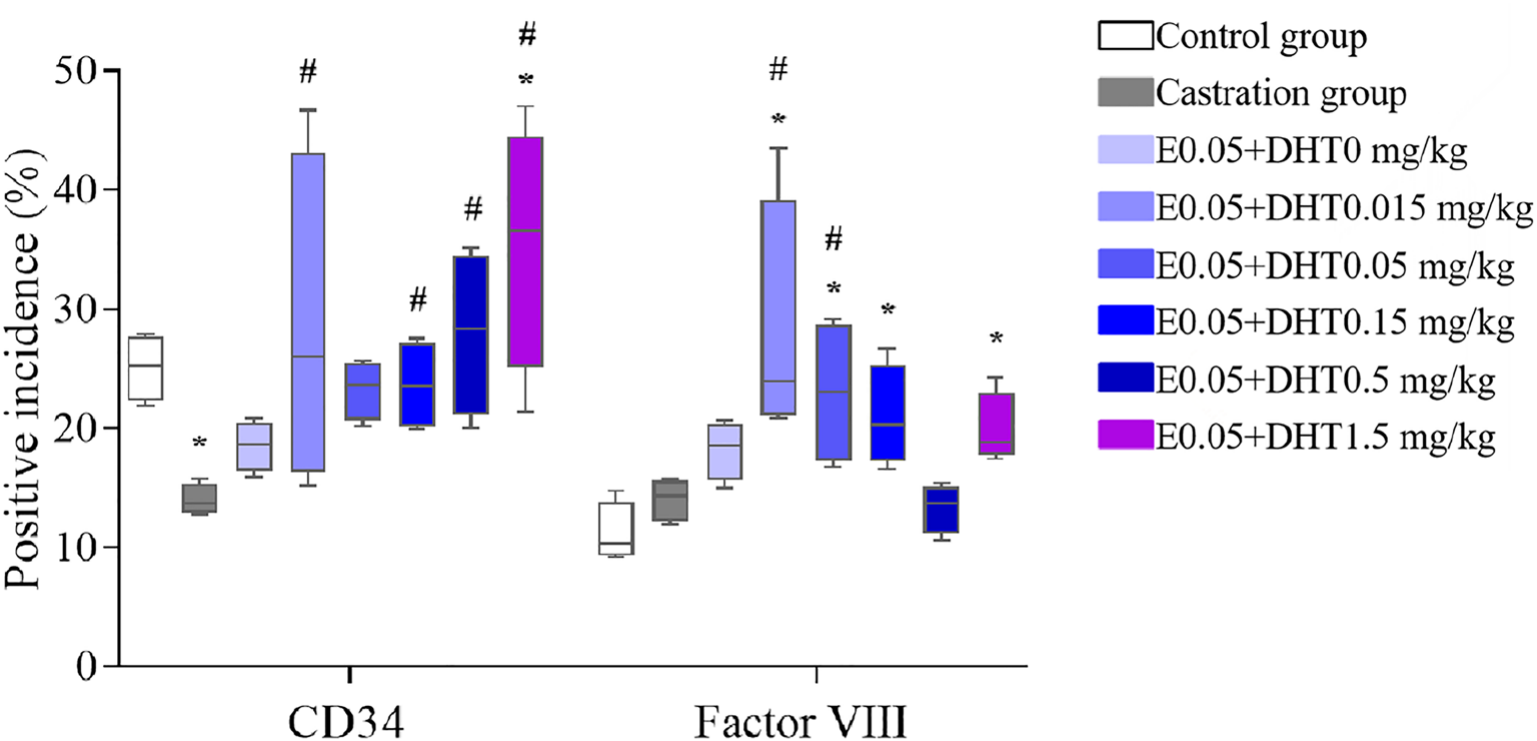
The positive incidences of CD34 and factor VIII in SD rats under the different concentrations of DHT. The data are shown as box-and-whisker plots. The plots show the minimum and maximum values (lower and upper horizontal black lines, respectively), the mean value (in black), the H-spread values (mean ± 25%, box). Nonparametric tests were used to analyse the data (*n* = 4). *P < 0.05 compared to control group, ^#^*P* < 0.05 compared to castration group

When the exogenous E concentration was constant (Fig. 5), except for the E0.05 + DHT0.015 mg/kg group, the positive rate of CD34 gradually increased with increasing exogenous DHT concentration in the E0.05 + DHT0 mg/kg, E0.05 + DHT0.05 mg/kg, E0.05 + DHT0.15 mg/kg, E0.05 + DHT0.5 mg/kg, and E0.05 + DHT1.5 mg/kg groups. Among them, the E0.05 + DHT1.5 mg/kg group was significantly different from the control group (P < 0.05). The differences between the E0.05 + DHT0.15 mg/kg, E0.05 + DHT0.5 mg/kg and E0.05 + DHT1.5 mg/kg groups were statistically significant (*P* < 0.05) compared with the castration group. However, there was no significant linear relationship between the positive rate of factor VIII expression and DHT concentration; the positive rates were significantly increased in the E0.05 + DHT0.015 mg/kg, E0.05 + DHT0.05 mg/kg, and E0.05 + DHT0.15 mg/kg groups compared with the control and castration groups (*P* < 0.05).

**Fig. 6:**
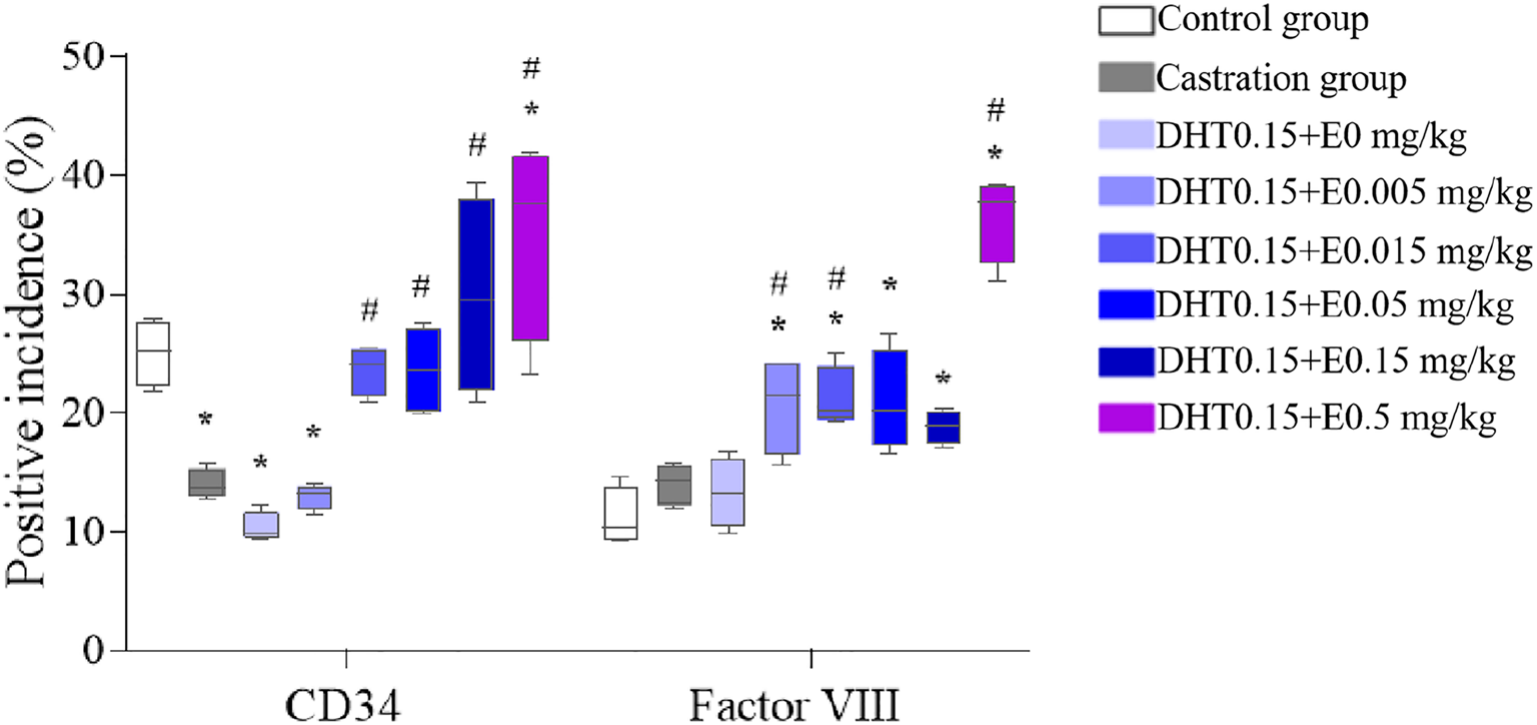
The positive incidences of CD34 and factor VIII in SD rats under the different concentrations of E. The data are shown as box-and-whisker plots. The plots show the minimum and maximum values (lower and upper horizontal black lines, respectively), the mean value (in black), the H-spread values (mean ± 25%, box). Nonparametric tests were used to analyse the data (*n* = 4). *P < 0.05 compared to control group, ^#^*P* < 0.05 compared to castration group

When the exogenous DHT was constant (Fig. 6), the positive rate of CD34 expression in each group gradually increased with increasing exogenous E concentration; among them, compared with the castration group, there appeared to be significantly higher positive rates in the DHT0.15 + E0.015 mg/kg, DHT0.15 + E0.05 mg/kg, DHT0.15 + E0.15 mg/kg, and DHT0.15 + E0.5 mg/kg groups, and the difference was statistically significant (*P* < 0.05). In addition, compared with the control and castration groups, the positive rates of factor VIII expression were significantly increased in the DHT0.15 + E0.005 mg/kg, DHT0.15 + E0.015 mg/kg, DHT0.15 + E0.05 mg/kg, and DHT0.15 + E0.5 mg/kg groups (*P* < 0.05).

### Prostatic MVD in each group

**Fig. 7:**
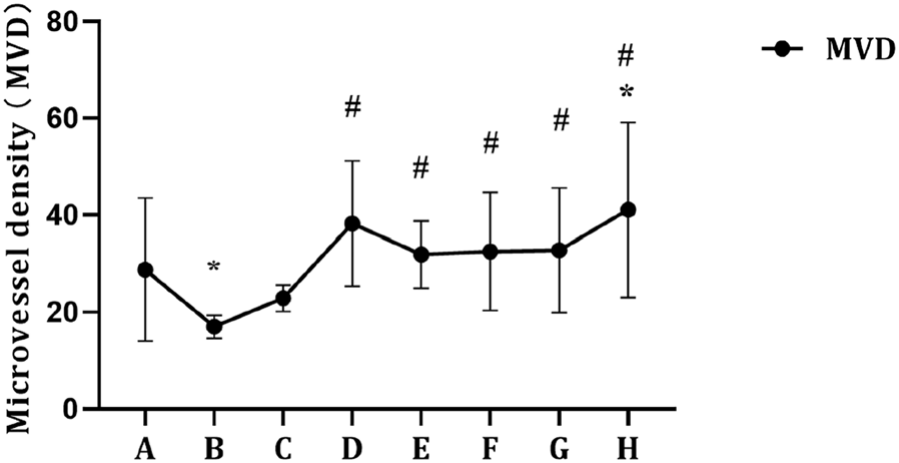
MVD in SD rats under the different concentrations of DHT. The data are shown as mean ± SD, and the statistical difference was determined using one-way analysis of variance (*n* = 4). *P < 0.05 compared to control group, ^#^*P* < 0.05 compared to castration group. **A** Control group. **B** Castration group. **C** E0.05 + DHT0 mg/kg group. **D** E0.05 + DHT0.015 mg/kg group. **E** E0.05 + DHT0.05 mg/kg group. **F** E0.05 + DHT0.15 mg/kg group. **G** E0.05 + DHT0.5 mg/kg group. **H** E0.05 + DHT1.5 mg/kg group

**Fig. 8:**
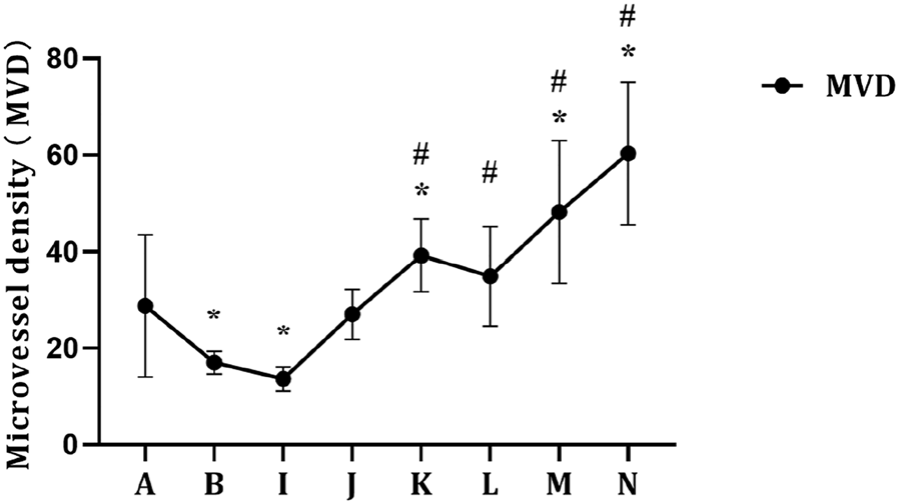
MVD in SD rats under the different concentrations of E. The data are shown as mean ± SD, and the statistical difference was determined using one-way analysis of variance (*n* = 4). *P < 0.05 compared to control group, ^#^*P* < 0.05 compared to castration group. **A** Control group. **B** Castration group. **I** DHT0.15 + E0 mg/kg group. **J** DHT0.15 + E0.005 mg/kg group. **K** DHT0.15 + E0.015 mg/kg group. **L** DHT0.15 + E0.05 mg/kg group. **M** DHT0.15 + E0.15 mg/kg group. **N** DHT0.15 + E0.5 mg/kg group

As shown in Figs. 7 and 8, compared with the control group, the MVD decreased significantly in the castration group (*P* < 0.05). When the exogenous E concentration was constant (Fig. 7), in general, the MVD of rats in all the groups increased with increasing exogenous DHT concentration. Compared with the castration group, the MVD increased significantly in the E0.05 + DHT0.015 mg/kg, E0.05 + DHT0.05 mg/kg, E0.05 + DHT0.15 mg/kg, E0.05 + DHT0.5 mg/kg, and E0.05 + DHT1.5 mg/kg groups (*P* < 0.05). In addition, when the exogenous DHT concentration was constant (Fig. 8), the MVD increased with increasing exogenous E concentration in all the groups. Among them, compared with the control and castration groups, the MVDs were significantly increased in the DHT0.15 + E0.015 mg/kg, DHT0.15 + E0.15 mg/kg, and DHT0.15 + E0.5 mg/kg groups (*P* < 0.05).

## Discussion

Benign prostatic hyperplasia is a prevalent disease among middle-aged and elderly men, and the exact pathogenesis remains unclear. At present, TURP is the gold standard for the surgical treatment of BPH [16], with less pain, less invasiveness, and faster recovery. However, intraoperative bleeding is still a common complication that can be life-threatening for patients. MVD is currently recognized as the standard for the evaluation of angiogenesis. Deering et al. [17] found that the prostate tissue of BPH patients may contain an "angiogenic switch" that can lead to an increase in MVD and that this angiogenic effect may begin in the early stages of BPH. Therefore, it is of great significance to strengthen the study of MVD for the prevention and treatment of perioperative bleeding in BPH patients.

The disruption of the balance between the concentrations of oestrogen and androgen is key to the occurrence and development of BPH [8]. Moreover, in our previous study, we found that the inflammatory response of the prostate in SD rats could be successfully induced by different concentrations of oestrogen and androgen [13]. Therefore, it is reasonable to hypothesize that the prostate, as a target organ of sex hormones, and the regulation of prostatic MVD may be associated with changes in oestrogen and androgen levels. In addition, the MVD may also change under the effect of different concentrations of oestrogen and androgen.

Finasteride, as a noncompetitive 5α-reductase inhibitor, inhibits the conversion of testosterone to DHT and reduces the concentration of DHT in vivo [18]. After preoperative administration of finasteride, a reduction in DHT concentration was found to be associated with a significant reduction in perioperative bleeding during TURP [19, 20]. This may be related to the reduction in DHT concentration, atrophy of the prostate gland, MVD, and lower blood flow after finasteride treatment [21–23]. In addition, it has been suggested that androgens can regulate vascular distribution in the prostate by regulating different growth factors, such as basic fibroblast growth factor and vascular endothelial growth factor [21, 24, 25]. Therefore, changes in androgen levels are essential for the regulation of prostate MVD. In this study, rats were induced by castration combined with different concentrations of oestrogen/androgen, and the results showed that the prostatic MVD in the castration group was significantly lower than that in the control group due to androgen source blocking. Some studies have demonstrated that testosterone acts as a stimulator of vascular endothelial growth factor, while androgen deprivation could lead to reduced blood flow to the prostate [26]. Furthermore, androgenic effects are mediated by vasoactive substances produced by dihydrotestosterone-regulated stroma and epithelial cells, which contain androgen receptors [27]. In this study, when the exogenous oestrogen concentration was constant, the prostate MVD of rats in each group increased with increasing exogenous DHT concentration, except for the E0.05 + DHT0.015 mg/kg group. Furthermore, we confirmed that androgens play an important role in regulating prostate MVD. The decrease in DHT concentration can cause a decrease in prostatic MVD. Conversely, prostatic MVD can be increased with increasing DHT concentration. Therefore, for patients with a large prostate volume scheduled for TURP, preoperative administration of noncompetitive 5α-reductase inhibitors to reduce the DHT concentration in vivo can help reduce the risk of perioperative bleeding.

In our clinical experience, we have found that BPH rarely occurs in young patients with relatively high androgen levels and low oestrogen levels and occurs more often in middle-aged and elderly patients with relatively low androgen levels and high oestrogen levels. Roberts et al. [28] found that in ageing males, serum androgen levels are decreased and oestrogen levels are relatively increased, which may be a vital reason for the development of BPH. Therefore, it is important to clarify the effect of changes in oestrogen levels on the MVD of prostate tissue undergoing TURP treatment in patients with enlarged prostates.

Oestrogen plays an important role in angiogenesis in a variety of tissues by increasing coronary blood flow and decreasing coronary resistance and peripheral vascular tension. In this sense, oestrogen could induce endothelial cell proliferation and migration and increase the expression of vascular endothelial growth factor [29]. In addition, Zhang et al. [30] studied changes in bone microvascular structure and vascular density after exogenous oestrogen supplementation in rats and found that the changes in oestrogen levels could affect the bone changes in MVD and that the formation of blood vessels in bone increases with an increased dose of oestrogen. Diedrich et al. [31] reported the effect of vulvovaginal atrophy and local oestrogen therapy on vaginal microcirculation structure. Using in vivo noninvasive techniques, oestrogen therapy was found to have a significant effect on vaginal microcirculation structure, and local oestrogen therapy can restore vaginal blood vessels.

At present, there is still a lack of relevant research on the effect of changes in oestrogen levels on the MVD of prostate tissue. This is the first systematic study of the effect of oestrogen/androgen changes on prostate MVD. We confirmed that androgens play an important role in the regulation of MVD in the prostate of SD rats. In addition, when SD rats were castrated, a certain concentration of exogenous DHT was supplemented to increase the oestrogen concentration. We first confirmed that the prostatic MVD can be increased with increasing oestrogen concentration. However, it is worth noting that the angiogenic effects of oestrogen are caused by several mechanisms. Oestrogen replacement therapy can increase the expression of vascular endothelial growth factor and its receptor [32], which regulates endothelial cell proliferation and permeability [33]. Additionally, at weeks 3 and 4 of oestrogen therapy, oestrogen can increase endothelial progenitor cells, directly stimulate their mitosis and migration activity, and inhibit apoptosis of endothelial progenitor cells [34, 35]. Furthermore, the angiogenic effects of oestrogen depend on the form, dose, and duration of administration, as well as on the cellular environment [36]. It is suggested that long-term oestrogen replacement therapy may increase the expression of vascular endothelial growth factor and endothelial progenitor cells, thereby increasing the synthesis, migration, and lumen formation of prostate microvascular endothelial cells. Without a doubt, it is necessary to confirm these findings by more comprehensive and systematic experiments in vivo and in vitro.

In addition, in this study, we found that the incidence of factor VIII positivity was different from that of CD34 positivity. These two endothelial cell antigens, namely, factor VIII and CD34, mark blood vessels of various maturities [15]. Anti-CD34, an antibody that is very sensitive to endothelial cell differentiation, stains tumour endothelial cells more strongly than normal endothelial cells [37]. Da Silva et al. [38] found that the MVD detected by anti-CD34 was significantly higher than that of anti-factor VIII in breast cancer tissues. However, there is a lack of systematic studies on whether there is a difference between CD34 and factor VIII in detecting MVD in nontumour tissues, especially in the prostate, which provides a new direction for our research in the next stage.

We also needed to point out the potential limitations in this study. First of all, although the selection of numbers in each group was not perfect, we found that the results were statistically significant during the analysis of the data. In addition, there was a linear relationship between prostatic MVD and different concentrations of oestrogen/androgen. Therefore, we believe that the results and conclusions still have good credibility. Secondly, we did not involve in vitro experiments such as cell culture experiment and so on. Therefore, it is still necessary to further strengthen the research in vitro in the future.

## Conclusions

The changes in oestrogen and androgen concentrations play an important role in the regulation of prostatic MVD in SD rats. A decrease in DHT concentration can induce a decrease in prostatic MVD, while prostatic MVD can be increased with increasing DHT concentration. Conversely, prostatic MVD increased gradually with increasing oestrogen concentration. Therefore, strengthening the monitoring and regulation of oestrogen and androgen levels in BPH patients during the perioperative period has certain guiding significance for reducing the risk of perioperative bleeding in BPH patients. Of course, these findings need to be further confirmed by more systematic and comprehensive research in the future.

## Data Availability

The datasets analysed during the current study are available from the corresponding author on reasonable request.
